# Individualized functional targeting for rTMS: A powerful idea whose time has come?

**DOI:** 10.1002/hbm.25543

**Published:** 2021-05-25

**Authors:** Fidel Vila‐Rodriguez, Sophia Frangou

**Affiliations:** ^1^ Non‐Invasive Neurostimulation Therapies (NINET) Laboratory, Department of Psychiatry University of British Columbia Vancouver British Columbia Canada; ^2^ Djavad Mowafaghian Centre for Brain Health University of British Columbia Vancouver British Columbia Canada; ^3^ Department of Psychiatry Icahn School of Medicine at Mount Sinai New York City New York USA

Transcranial magnetic stimulation (TMS) is a focal, non‐convulsive, non‐invasive neuromodulation technique that can electromagnetically induce currents strong enough to trigger action potentials. The activation of these focal cortical volumes leads to modulation of brain areas distal to the target stimulated. Therefore, TMS stimulates relatively focal cortical areas but modulates brain networks.

Neuropsychiatric disorders are increasingly conceptualized in terms of brain network dysfunction. Depression is a prime example, and the functional connectivity (FC) between the subgenual anterior cingulate cortex (SGC) and the dorsolateral prefrontal cortex (DLPFC) is has been identified as a key target to modulate (Goldapple et al., 2004). Indeed, the SGC‐DLPFC FC is a predictor of treatment response (Fox, Liu, & Pascual‐Leone, 2013), and it is correlated with clinical improvement (Ge, Downar, Blumberger, Daskalakis, & Vila‐Rodriguez, 2019).

In this regard, DLPFC targeting was initially based on methods that approximated the *structural* anatomical location of the DLPFC based on simple, pragmatic methods (i.e., heuristic). The first heuristic was to target the DLPFC by moving 5 cm anteriorly from the motor threshold hotspot location (the “the rule of 5 cm” method), which was subsequently modified to the “5.5 cm rule” and the “6 cm rule”. The most recent and established heuristic, the “Beam F3 method” was validated using high‐resolution anatomical MRI scans and provides a feasible and reliable clinical use method (Beam, Borckardt, Reeves, & George, 2009). In parallel, precision targeting systems (i.e., neuronavigation system) allow the use of individual MRI head scans to be registered in space with fiduciary markers. These systems achieve precise and consistent millimeter targeting of brain areas and facilitate target consistency across treatment sessions. This precise structural anatomical targeting facilitated the definition of *group‐level functional* targeting based on averaged coordinates where the SGC‐DLPFC was maximum (Fox et al., 2013).

In this issue of the journal (Cash et al., 2021), Cash and colleagues provide the foundation to move the field from group‐level functional targets to *individualized functional targets* (iFT). Using the Human Connectome Project public dataset, they computed FC maps between a DLPFC ROI (comprising BA9, BA46, a volume around the “rule of 5 cm” target and the Beam F3 target) and the SGC using the conventional seed‐based approach (i.e., a 10 mm radius spherical mask centered at MNI 6, 16, −10) and a seedmap approach (i.e., using a weighted spatial average of the fMRI data across all gray matter voxels). Subsequently, they compared three different computation methods to derive an iFT, namely “classic” (i.e., select the single most anticorrelated voxel), “searchlight” (computing SGC FC within half‐spheres at each voxel within the DLPFC ROI), and “cluster” (i.e., retaining only a specified portion of the most anticorrelated voxels and spatially cluster). The metrics used to quantify each approach's reliability were the distance of the iFT between two sessions in the same person (i.e., intraindividual distance) and the distance of the iFT between different persons (i.e., interindividual distance). Parameters investigated for optimization included the cluster size, degree of spatial smoothing and scanning time. Last, they investigated whether iFT was genetically driven and stable over 1 year.

The results show a high degree of robustness as the intraindividual distance between scan sessions was a median of 2.2 mm while showing that there is individual heterogeneity in the location of the iFT with targets scattered broadly across the DLPFC and a median interindividual distance between 16 and 27 mm. The combination of the cluster and seedmap methodology showed the best performance, and interestingly the optimal scanning time was estimated at ~20 min. The iFT localization showed a pattern consistent with some level of genetic influence and the iFT was relatively stable over in a subgroup of subjects who underwent a second scan within a year.

The present work shows the robustness and feasibility of a method to derive iFT using resting state‐fMRI and thus provides investigators with a valuable tool to investigate further questions. The area that this tool may be applied to is certainly in treatment of depression with rTMS and as it might tackle several related clinical questions. First, a clinical trial to investigate the efficacy of iFT vs. other targeting method(s) maybe warranted and if the hypothesis that using iFT is superior holds it may lead to improvements in current outcomes of rTMS for depression. Second, an iFT approach would allow to interrogate the question of what proportion of depression cases are primarily driven by abnormal SGC‐DLPFC connectivity and in those who do not respond to a DLPFC‐iFT, perhaps contemplate a systematic investigation of alternate brain targets (e.g., dorsomedial prefrontal cortex (Dunlop et al., 2020)). Third, although the iFT seems stable over time in healthy volunteers, the question of whether the course of rTMS is associated with changes in the iFT will be important to address.

The electric field (e‐field) induced by TMS stimulates tissue that is geometrically aligned with it, and modeling work estimates that the crown and lips of gyri are the cortical aspects where the e‐field induced by most TMS coils is optimal. The e‐field can be modeled at the individual level using MRI and thus providing a fairly accurate estimation of how the coil needs to be oriented to elicit an optimal stimulation. Indeed, recent work has combined task based (working memory) functional targeting of DLPFC with individualized electric field modeling which represents another layer of refinement and precision (Balderston et al., 2020). Similarly, it would have been informative to understand the impact of the individualized e‐field on the iFT in the present work; this will be an aspect that warrants further investigation.

In closing, Cash and colleagues have developed a tool that shows robust performance to be used to address relevant questions. This work along with concurrent efforts to develop similar iFT for other conditions such as Alzheimer's disease or Schizophrenia may be signaling that psychiatry may be ready to embark in an era of precision medicine.
